# Prognostic role of Gli1 expression in breast cancer: a meta-analysis

**DOI:** 10.18632/oncotarget.19080

**Published:** 2017-07-07

**Authors:** Bilan Wang, Ting Yu, Yuzhu Hu, Mengmeng Xiang, Haoning Peng, Yunzhu Lin, Lu Han, Lingli Zhang

**Affiliations:** ^1^ Department of Pharmacy, West China Second University Hospital, Sichuan University, Chengdu, 610041, PR China; ^2^ Evidence-Based Pharmacy Center, West China Second University Hospital, Sichuan University, Chengdu, 610041, PR China; ^3^ Key Laboratory of Birth Defects and Related Diseases of Women and Children, Ministry of Education, West China Second University Hospital, Sichuan University, Chengdu, 610041, PR China; ^4^ Cancer Center, West China Hospital, West China Medical School, Sichuan University and Collaborative Innovation Center, Chengdu, 610041, PR China

**Keywords:** Gli1, breast cancer, prognosis

## Abstract

Glioma-associated oncogene 1 (Gli1) is a critical transcriptional factor of Sonic hedgehog pathway which has been proved to participate in the initiation and progression of tumor in mammalians. However, its clinical value in breast cancer remains unknown. Thus, a meta-analysis was performed to clarify the association of Gli1 over-expression, clinic-pathological characteristics, molecular subtypes and prognosis in breast cancer. According to included criteria, 13 eligible studies containing 2816 patients all around the world were selected in this study. Our results indicated no significant association of Gli1 expression and histological grade (RR = 1.20, 95% CI: [0.98, 1.47]), T stage (RR = 1.05, 95% CI: [0.87, 1.27]), clinical stage (RR = 1.04, 95% CI: [0.93, 1.18]) and lymph node metastasis (RR = 1.12, 95% CI: [0.92, 1.37]). In addition, pooled RR showed no correlation of Gli1 expression and progesterone receptor (PR) (RR = 0.92, 95% CI: [0.70, 1.21]), estrogen receptor (ER) (RR = 1.03, 95% CI: [0.74, 1.42]), human epidermal growth factor receptor 2 (HER-2) (RR = 1.12, 95% CI: [0.90, 1.39]). Nonetheless, up-regulated Gli1 expression predicts shorter disease-free survival (DFS) (HR = 1.38, 95% CI: [1.05, 1.81]), 3-year survival (HR = 1.74, 95% CI: [1.28, 2.36]), 5-year survival (HR = 2.04, 95% CI: [1.62, 2.57]) and overall survival (OS) (HR = 2.05, 95% CI: [1.60, 2.64]). In conclusion, over-expression of Gli1 tends to progressive stages and is related to unfavorable prognosis of breast cancer, which may become a potential prognosis indicator and therapy target in breast cancer.

## INTRODUCTION

Worldwide, morbidity of breast cancer has been increased to 1.7 million in 2012. For females, breast cancer has the highest mortality rate among all cancer types, accounting for 15% of all cancer deaths [1]. In China, its age-standardized incidence rate has been increasing significantly in the last decade [2]. The general therapeutic strategies are based on locoregional tumor load, molecular subtype and patients’ preferences in early breast cancer [3]. In metastatic breast cancer, principles of systemic therapy mainly include chemotherapy, targeted therapy and endocrine therapy [4]. Breast cancer biology plays an important role in the selection of therapeutic plan [5]. Moreover, progesterone receptor (PR), estrogen receptor (ER) and human epidermal growth factor receptor 2 (HER-2) are the only clinically relevant biomarkers and verified therapeutic targets in metastatic breast cancer. However, potential heterogeneity between primary tumor and metastasis, even between metastases, find a potential therapeutic target becomes particularly important, especially in triple-negative metastatic breast cancer [6, 7]. Therefore, more effective therapeutic strategies of breast cancer underlie a better understanding of novel molecular targets and signaling pathways that are closely related to the clinic-pathological prognostic factors of breast cancer.

Sonic hedgehog (Shh) signaling pathway, one of the components of the hedgehog (hh) pathway, was originally known to play an important role in embryonic development, cell maturation including differentiation, proliferation and maintenance of tissue polarity [7–9]. Recent studies have demonstrated that the SHH pathway was also involved in the invasion and metastasis process of solid tumor via its interaction with cancer stem cells (CSC) [10, 11] This signaling pathway is initiated with the secretion of Shh glycoprotein, activating the transmembrane protein Patched 1 (PTCH1) by binding with it. The activation of the PTCH1 relieves the inhibition of the Smoothened (Smo), thereby leading to the activation of Glioma-associated oncogene 1 transcription factors [7]. As the final effective factor of the Shh pathway, Gli1, which is considered as a valuable maker of Shh pathway activation, has been reported to be involved in cell self-renewal, cell proliferation, survival, invasion, angiogenesis and epithelial-mesenchymal transition through regulating expressions of certain genes [12, 13]. Thus, abnormal cell development and even oncogenesis tend to happen when Gli1 is dysregulated. Several types of carcinoma have been reported to have aberrant activation of Gli1, including hepatocellular carcinoma, gastric cancer, lung cancer, breast cancer and basal cell carcinoma, indicating the dysregulation of Gli1 may contribute to malignant biological behavior [14–16]. Clinical studies have also demonstrated that the expression of Gli1 can be utilized as a potential maker and imply poor prognosis in lung squamous cell carcinoma, basal cell carcinoma, head and neck squamous carcinoma which indicting the potential prognostic value of Gli1 [17, 18]. However, the functional and prognostic significance of Gli1 in breast cancer still remains unclear. Some studies have indicated that over-expression of Gli1 predicted poor outcome of breast cancer with higher tumor stage and increased number of tumor-positive axillar lymph nodes [19, 20]. While other study showed no significant correlation between expression level of Gli1 and cancer-specific survival in ERα-positive breast cancer [20].

Hence, to clarify the association of Gli1 over-expression and clinicopathological features, molecular subtypes, and clinical outcomes in breast cancer, a meta-analysis was performed via acquired available published data.

## RESULT

### Literature selection and characteristics

**Figure 1:**
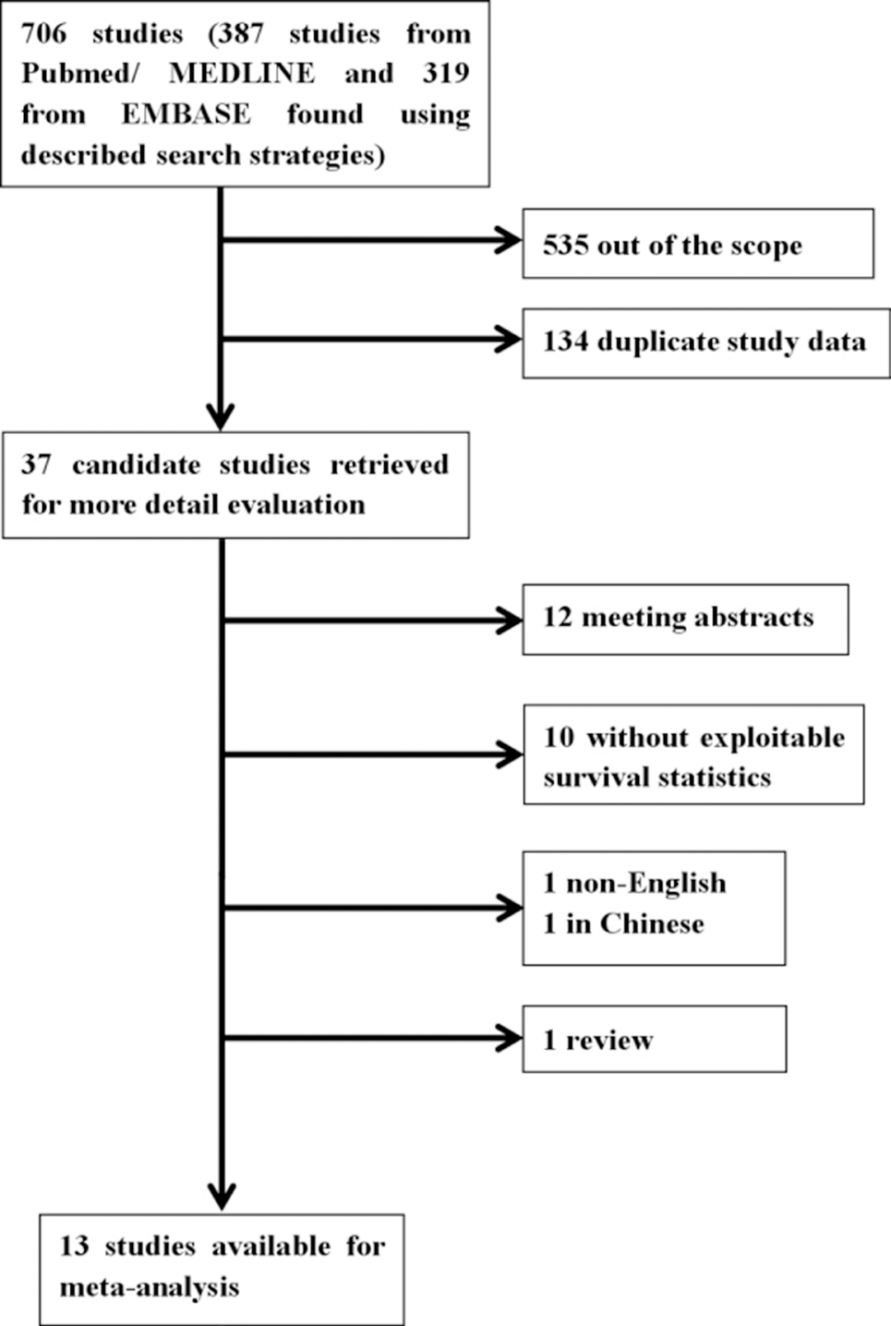
Flow chart of the literature search of this meta-analysis

We recruited 706 eligible studies from Pubmed/MEDLINE (*n* = 387) and EMBASE (*n* = 319) according to the literature retrieval method mentioned above. Two independent investigators (Bilan Wang and Ting Yu) went through title and abstract of these articles and excluded 535 unrelated citations. Meanwhile, 134 studies were removed from scope because of duplicate data. The remaining 37 candidate studies were reviewed carefully by full text. Among them 12 were conference abstracts and 10 were not exploitable for survival data or clinic-pathological data associated with Gli1. In addition, 1 non-English (Chinese) and 1 review article were excluded as well. Ultimately, our study included 13 articles for further data extraction and analysis (Figure 1). The 13 included studies [8, 19, 20, 23–32] were published between 2009 and 2016. A total number of 2816 patients from Australia, America, Sweden, China, Korea and Germany were investigated and sample size varied from 83 to 334. All studies performed immunohistochemistry (IHC) to evaluate the expression of Gli1. In data analysis, 6 studies [8, 19, 25, 29, 31, 32] provided information for overall survival (OS), 4 studies [8, 25, 31, 32] provided disease free survival (DFS), 1 study [20] provided cause specific survival (CSS), 1 study [23] provided distal metastasis free survival (DMFS), 1 study [27] for regression free survival (RFS), 1 study [28] for event free survival (EFS). Detailed Characteristics were demonstrated in Table 1, and main outcomes presented in this meta-analysis were summarized in Table 2.

**Table 1:** Characteristics of all identified studies

	first author (ref)	year	country	number (F)	pathological classification	molecular classification	Surgery(%)	method	antibody source	definition of Gil+ positive	expression rate (%)	median follow-up (m)	survival analysis	NOS score
1	Sandra A. O'Toole	2011	Australia	282	invasive ductal carcinoma/ ductal carcinoma in situ	NR	NR	IHC	Santa Cruz	≥20%	31%	NR	OS	7
2	Lusheng Xu	2010	USA	171	infiltrating ductal carcinomas	NR	NR	IHC	Santa Cruz	＞2%	32.37%	93.6	CSS	7
3	Yumei Diao	2016	Sweden	126	NR	ERα-positive	NR	NR	NR	NR	NR	NR	DMFS	6
4	Shiwei Liu	2016	China	108	IBC	HER2-positive	100%	IHC	Abcam	IRS score≥7	39.81%	32	EFS、PFS	9
5	Bhuvaneswari Ramaswamy	2012	U.S.	289	IBC	NR	NR	IHC	Sigma	≥10%	Epithelial 69%, stromal 49%	96	OS、DFS	9
6	Yuan Li	2012	China	262	IBC	All	100%	IHC	Santa Cruz	score≥2*	33.59%	48.2	OS、DFS	7
7	Miao He	2015	China	290	NR	NR	100%	IHC	Abcam	IRS score≥3	54.80%	NR	OS、DFS	10
8	Haishan Zhao	2016	China	266	Invasive ductal carcinoma/ invasive lobular carcinoma	NR	100%	IHC	Abcam	IRS score≥3	52.60%	48-77	OS、DFS	8
9	Anette ten Haaf	2009	Germany	204	IBC	NR	100%	IHC	Santa Cruz	IRS score≥7	97%	78	OS	10
10	Yinghua Li	2012	China	284	ductal and lobular cancer	NR	100	IHC	Santa Cruz	IRS score≥7	83.10%	62	RFS	9
11	Soyoung Im	2013	Korea	334	IBC	All	100	IHC	Abcam	IRS score≥4	42.20%	75.1	NR	8
12	Yajun Tao	2011	China	83	invasive ductal carcinomas/ invasive lobular carcinomas	All	NR	IHC	Santa Cruz	NR	NR	NR	NR	10
13	Xin Ge	2015	China	117	NR	NR	NR	IHC	Santa Cruz	IRS score≥5	NR	NR	NR	8

NR, not reference; IBC, invasive breast cancers; IHC, immunohistochemistry; IRS, immuno-reactive-score; DFS, disease free survival; OS, overall survival; CSS, cause free survival; EFS, event free survival; RFS, recurrence free survival; DMFS, distant metastasis free survival; NOS, Newcastle-Ottawa scale.

The nuclear staining intensity: 0 and 3 (0 = negative, 1 = weak, 2 = moderate, and 3 = strong), the percentage of nuclear stained cells: 0 and 4 (1 ≤25%, 2 = 26−50 %, 3 = 51−75%, and 4 > 75%). IRS was calculated by multiplication of the two scores

*The expressions of Glil was scored as intensity of staining: 0(no staining), 1 (weak/moderate staining) or 2 (intense staining). Glil nuclear over-expression was identified for intensity cases with staining intensity of 2+.

**Table 2:** Summary of the outcomes presented in this meta-analysis

Group	No. of studies	No. of total patients	RR/HR (95% CI) (Gli1 positive VS Gli1 negative)	*P* for heterogeneity	I^2^	references
Histological grade	7	1374	1.20 (0.98, 1.47)	0.013	62.9%	[19, 24, 26, 28, 30–32]
T stage	7	1309	1.05 (0.87, 1.27)	0.051	52.1%	[19, 24, 26–28, 30, 32]
Clinical stage	3	735	1.04 (0.93, 1.18)	0.371	0.0%	[24, 26, 27]
Lymph node metastasis	8	1658	1.12 (0.92, 1.37)	0.000	75.9%	[19, 24, 26–28, 30–32]
ER	5	1366	1.03 (0.74, 1.42)	0.000	88.0%	[19, 26, 27, 32]
PR	4	1084	0.92 (0.70, 1.21)	0.000	83.8%	[19, 26, 27, 29, 32]
Her-2	3	880	1.12 (0.90, 1.39)	0.106	55.4%	[26, 27, 32]
DFS	4	1107	1.48 (1.14, 1.93)	0.038	64.5%	[8, 25, 31, 32]
3-year survival	6	1593	1.74 (1.28, 2.36)	0.278	20.6%	[8, 19, 25, 29, 31, 32]
5-year survival	6	1593	2.04 (1.62, 2.57)	0.482	0.0%	[8, 19, 25, 29, 31, 32]
OS	6	1593	2.10 (1.64, 2.68)	0.961	0.0%	[8, 19, 25, 29, 31, 32]

### Gli1 expression and clinic-pathological parameters

**Figure 2:**
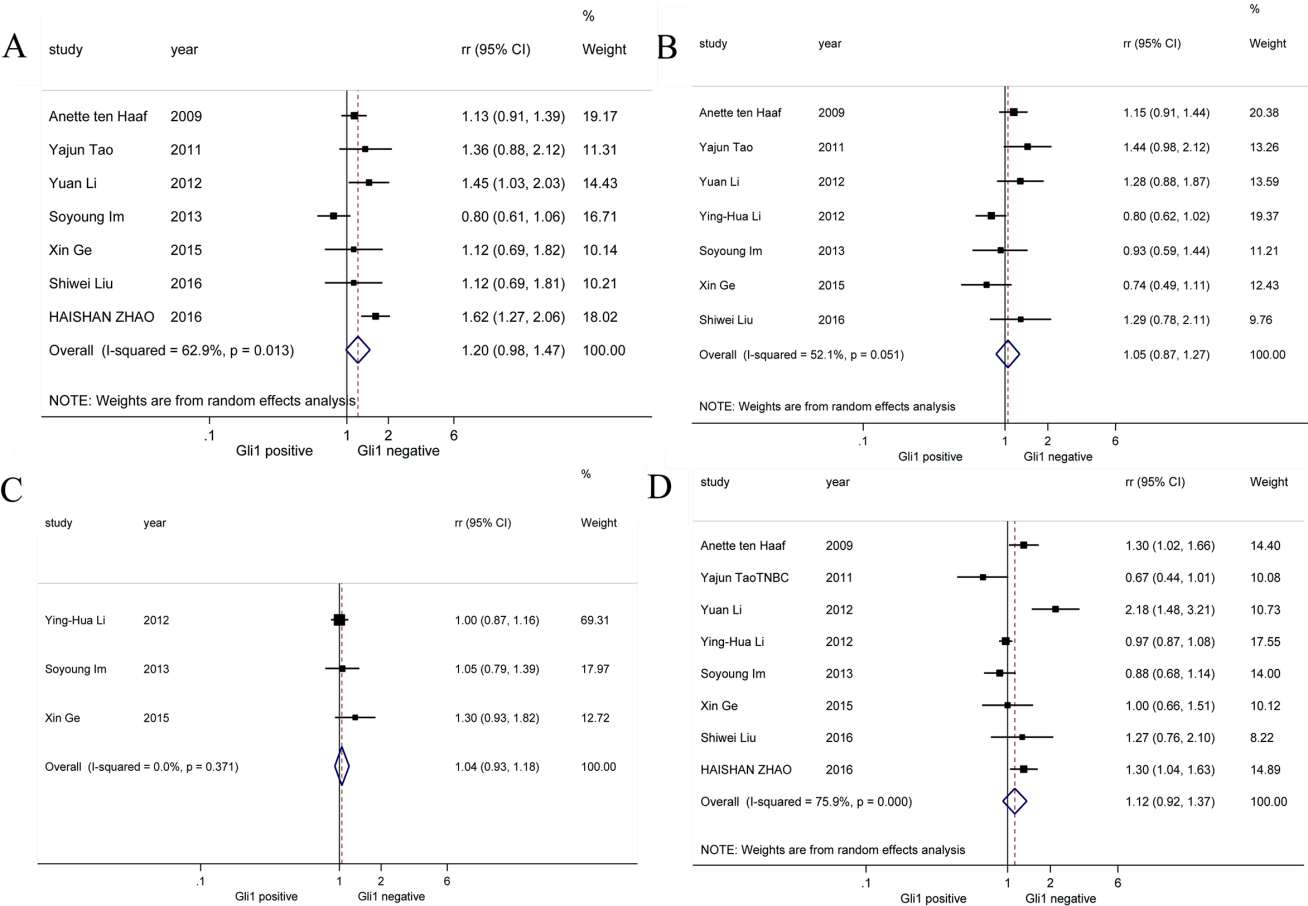
Forrest plots of RRs for correlation between Gli1 over-expression and clinicopathological features (**A**) Histological grade, (**B**) T stage, (**C**) clinical stage and (**D**) lymph node metastasis.

We performed pooled analysis on correlations between Gli1 expression and a series of clinic-pathological parameters (Figure 2). Firstly, we summarized data about histological grade and Gli1 expression from 7 studies [19, 24, 26, 28, 30–32] and discovered that histological grade was not correlated with Gli1 high expression (RR = 1.20, 95% CI: [0.98, 1.47]). Next, data on T stage and Gli1 expression from another 7 studies [19, 24, 26–28, 30, 32] were calculated and we found that there is no significant association between T stage and Gli1 expression (RR = 1.05, 95% CI: [0.87, 1.27]). Then, we investigated 3 studies [24, 26, 27] finding no obvious correlation between clinical stage and Gli1 high expression (RR = 1.04, 95% CI: [0.83,1.18]). Finally, lymph node metastasis was not associated with Gi1 expression based on information from 8 studies [19, 24, 26–28, 30–32] (RR = 1.12, 95% CI: [0.92,1.37]). For histological grade, T stage and lymph node metastasis parameter analysis, random-effects model was utilized because of obvious heterogeneities (*I*^2^ = 62.9%, *I*^2^ = 52.1%, *I*^2^ = 75.9%, respectively), while fixed-effects model was applied in clinical stage parameter analysis (*I*^2^ = 0.0%). To sum up, our study revealed Gli1 expression was not associated with histological grade, T stage, clinical stage or lymph node metastasis in breast cancer.

### Gli1 expression and immunohistochemical parameters

**Figure 3:**
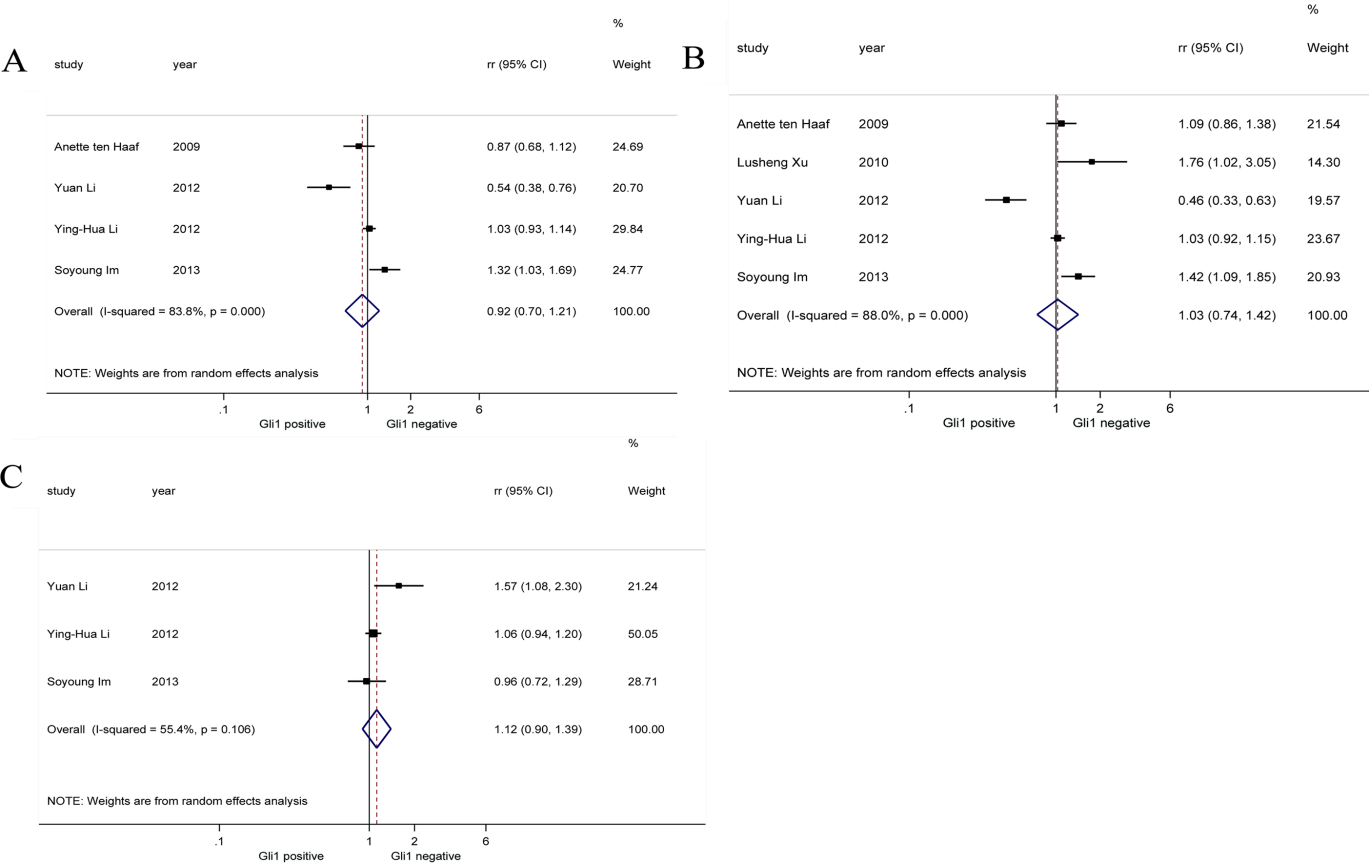
Forrest plots of RRs for correlation between Gli1 over-expression and immunohistochemical parameters in breast cancer (**A**) Progesterone receptor (PR), (**B**) oestrogen receptor (ER), and (**C**) human epidermal growth factor receptor 2 (HER-2).

Considering breast cancer is a typical hormone-related cancer, we performed pooled analysis on correlations between Gli1 expression and three specific immunohistochemical parameters (PR, ER, HER-2) in breast cancer patients (Figure 3). Our results reported there was no significant association between PR [19, 26, 27, 32] (RR = 0.92, 95% CI: [0.70, 1.21]), ER [19, 26, 27, 29, 32] (RR = 1.03, 95% CI: [0.74, 1.42]), HER-2 [26, 27, 32] (RR = 1.12, 95% CI: [0.90, 1.39]) expression and Gli1 expression. Random-effects model was applied in all analysis of correlation between PR, ER, HER-2 and Gli1 for obvious heterogeneities (*I*^2^ = 83.8%, *I*^2^ = 88.0%, *I*^2^ = 55.4%, respectively).

### Gli1 expression and breast cancer survival outcome

**Figure 4:**
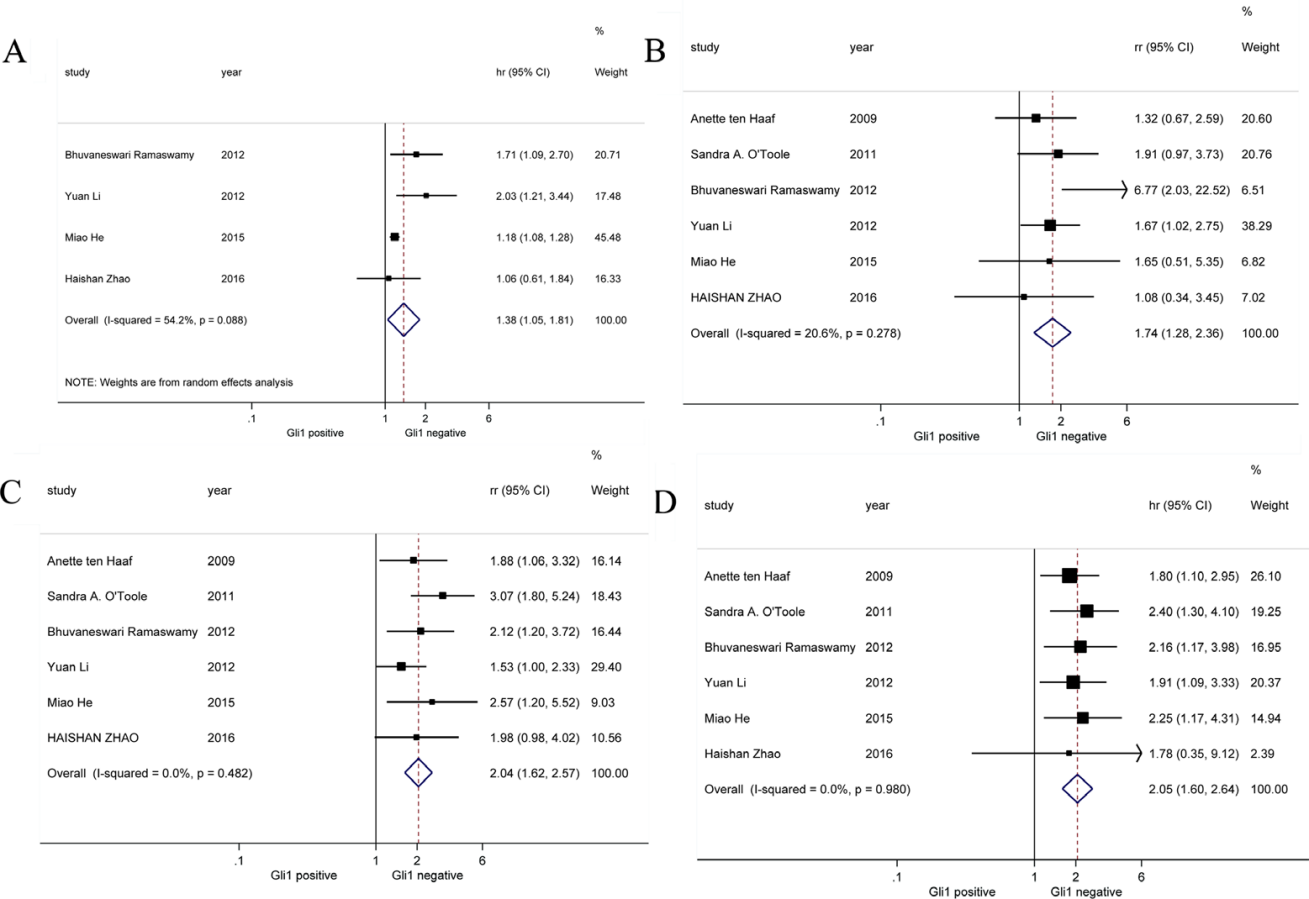
Forrest plots of HR for Gli1 over-expression and the clinical survival outcomes (**A**) DFS, (**B**) 3-year survival, (**C**) 5-year survival and (**D**) OS.

Survival outcomes of breast cancer with high and low Gli1 expression from 6 studies were extracted and analyzed (Figure 4). Result showed Gli1 over-expression was correlated with shorter DFS in breast cancer patients (HR = 1.38, 95% CI: [1.05, 1.81]). Moreover, both 3-year survival (HR = 1.74, 95% CI: [1.28, 2.36]) and 5-year survival (HR = 2.04, 95% CI: [1.62, 2.57]) were worse in high Gli1 expression breast cancer cohort compared with low Gli1 expression cohort. Consistently, there was also significant association between Gli1 over-expression and poor OS (HR = 2.05, 95% CI: [1.60, 2.64]) of breast cancer patients. Random-effects model was utilized in this analysis of DFS for obvious heterogeneity (*I^2^* = 54.2%). In all, our results indicated up-regulated expression of Gli1 was associated with poor survival outcomes involving DFS, 3-year survival, 5-year survival and OS.

### Publication bias

**Figure 5:**
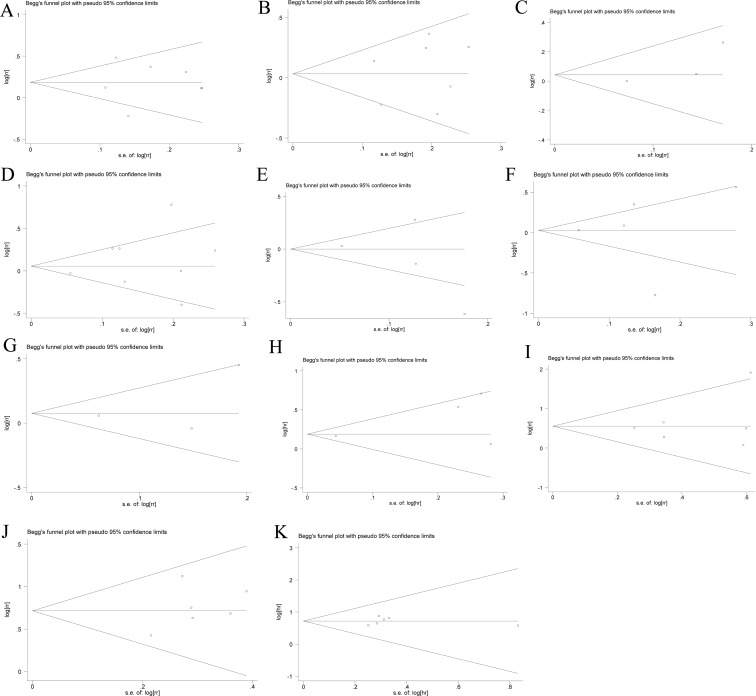
Funnel graph of assessing the potential publication bias of this study (**A**) Histological grade, (**B**) T stage, (**C**) clinical stage, (**D**) lymph node metastasis, (**E**) PR, (**F**) ER, (**G**) HER-2, (**H**) DFS, (**I**) 3-year survival, (**J**) 5-year survival and (**K**) OS.

Both Begg's funnel plot and Egger's test were performed to evaluate the publication bias in all studies assessing histological grade, T stage, clinical stage, lymph node metastasis, DFS, 3-year and 5-year survival and OS, respectively (Figure 5). Neither Begg's funnel plot nor Egger's test demonstrated any evidence of statistically significant asymmetry in the meta-analysis of histological grade (Begg: *p* = 0.881, Egger: *p* = 0.994), T stage (Begg: *p* = 0.881, Egger: *p* = 0.678), clinical stage (Begg: *p* = 0.117, Egger: *p* = 0.350), lymph node metastasis (Begg: *p* = 1.000, Egger: *p* = 0.430), PR (Begg: *p* = 0.174, Egger: *p* = 0.545), ER (Begg: *p* = 0.624, Egger: *p* = 0.998), HER-2 (Begg: *p* = 0.602, Egger: *p* = 0.660), DFS (Begg: *p* = 0.497, Egger: *p* = 0.0.304), 3-year survival (Begg: *p* = 0.348, Egger: *p* = 0.537), 5-year survival (Begg: *p* = 0.573, Egger: *p* = 0.312) and OS (Begg: *p* = 0.348, Egger: *p* = 0.934).

## DISCUSSION

Gli1 overexpression is revealed to have a close connection with breast cancer as a significant maker of aberrant activation of SHH pathway [33–35]. It involves in the formation and progression of breast cancer in many important ways such as activating tumor associated target genes [36, 37], promoting mammary epithelial cell mesenchymal transition (EMT) [38], regulating mammary CSC self-renewal [10] and facilitating angiogenesis [39]. In addition, researchers have confirmed that inhibiting the Gli1 expression could effectively attenuate tumor growth and migration and indicated its potential role as a therapeutic target in breast cancer management [40, 41]. However, its clinical value as a prognostic marker remains unclear due to differences in detective methods of Gli1 and selected patients, etc among researches. Therefore we carried out a meta-analysis based on available evidences in order to investigate Gli1 expression with survival and several clinic-pathological charactereristics in breast cancer patients.

Our study was the first one to summarize researches of prognostic role of Gli1 in breast cancer. The result indicated no significant association between Gli1 expression and several clinic-pathological characteristics such as histological grade, T stage, clinical stage and lymph node metastasis. Moreover, we reported PR, ER and HER-2 were not correlated with Gli1 expression in breast cancer. But the relationship between Gli1 over-expression and survival in breast cancer patients might be influenced by the clinic-pathological characteristics. Nevertheless, it was remarkable that we confirmed that breast cancer patients with over-expression of Gli1 tended to obtain a worse survival outcome referring to DFS, 3-year survival, 5-year survival and OS.

Diao's study [23] clarified that Gli1 would be a potential therapeutic target, moreover, could also act as a prognostic marker in breast cancer. The research observed that high Gli1 expression was associated with poor distant metastasis free survival (DMFS) in 126 patients (HR = 4.87, 95% CI: [1.34, 17.67]). So the clinical staff should pay more attention to patients with breast cancer with high Gli1 expression in systemic screening, such as increasing the frequency of bone scan. Li's study [27] showed that Gli1 expression is significantly correlated with aggressive features and unfavorable recurrence free survival (RFS). The breast cancer with nuclear Gli1 over-expression signified early relapse after radical operation, therefore mammary gland color ultrasound is particularly necessary in patient reexamination. Moreover, summarized DFS in our study also supported that the regular rechecks are more necessary in postoperative patients with Gli1 over expression. What's more, our results reported no significant association between Gli1 expression and hormone receptors expression, which is controversial with Makoto Kubo and Sun reported that Gli1 expression is positively correlated with ER expression [42, 43]. Further researches are required to clarify relationship between Gli1 and hormone receptors expression.

There still exist some limitations in our study, which deserve attention. Firstly, definitions of Gli1 over-expression, detection of subcellular Gli1 localization and antibody source varied in different studies. Secondly, only part of recruited patients has undergone surgery which may contribute to potential bias. Thirdly, for the reason that the number of eligible studies was limited, further detailed analysis of correlation between Gli1 over-expression and clinicopathological features hadn't been carried out. In addition, the heterogeneities across articles cannot be ignored.

Our meta-analysis is the first one to explain Gli1 expression as an aggressive biological behavior in breast cancer patients. It integrated convincing evidence to elucidate relationship between Gli1 expression and prognosis of breast cancer. The over expression of Gli1 could predict poor survival in patients with breast cancer. Moreover, detection of Gli1 can provid more convincing evidences for guiding the diagnosis and treatment in breast cancer patients.

## MATERIALS AND METHODS

### Literature retrieval

We retrieved articles from PubMed/Medline and EMBASE electronic databases by variablely combining the following terms “Glioma associated oncogene 1”, “Gli1”, “Hh”, “breast cancer”, “breast carcinoma”. The search ended on 10th March, 2017. Meanwhile, the references for articles and reviews were also screened to identify additional relevant articles. This meta-analysis included publications meeting the following criteria: (1) patients had a diagnosis of breast cancer; (2) expression level of Gli1 were determined with method such as IHC or reverse transcription-polymerase chain reaction (RT-PCR); (3) the studies provided direct hazard ratio (HR) and 95% confidence interval (CI) for survival or Kaplan-Meier survival curves at different Gli1 expression level; (4) the studies reported Gli1 expression according to different clinic-pathological parameters of patients. When there is possible duplication of data in several publications, we prefer the most recent or the most integrated study. Studies of letters, case reports, conference abstracts, editorials and reviews without original data; non-English papers; animal or laboratory studies were not in our scope.

### Data extraction

Two investigators reviewed eligible studies to obtain the following information: first author's name, year of publication, source of patients, sample size, mean age of patients, assay method, cut-off definition, TNM stage, histological grade, immunohistochemical features (ER, PR, HER-2), Gli1 expression rate and survival data according to Gli1 expression. Disagreements were discussed by the two investigators and settled by consulting a third author to reach consensus. Moreover, quality assessments of all studies included were conducted by Newcastle-Ottawa-Scale (NOS) criteria. High quality studies refer to those scored 5 or above 5.

### Statistical analysis

RevMan 5.0 software (Copenhagen, Denmark) was applied to analyze extracted data and calculate RR. To investigate the impact of Gli1 expression on survival outcome, HR and 95% CI from multivariate Cox hazard models were used. If direct data were not available, then Kaplan-Meier survival curves analyses were adopted to yield survival data as what Tierney demonstrated [21]. Further work on association between Gli1 and clinic-pathological characteristics (histological grade, T stage, clinical stage, lymph node metastasis), Gli1 and immunohistochemical parameters (PR, ER, HER-2), Gli1 and breast cancer survival (DFS, 3-year survival, 5-year survival, OS) were calculated by STATA 12.0 software (Stata Corporation, College Station, TX, USA) and presented in forest plots together with RR or HR and 95% CI, respectively. For studies with high heterogeneity (*I*^2^ > 50%) or *P* < 0.10, random-effects model was utilized for data analysis, otherwise the fixed-effects model was utilized. Publication bias was evaluated by funnel plot with a Begg's test [22]. Asymmetrical distribution of results was estimated to existing publication bias.
